# When Long Noncoding RNAs Meet Genome Editing in Pluripotent Stem Cells

**DOI:** 10.1155/2017/3250624

**Published:** 2017-11-23

**Authors:** Fuquan Chen, Jiaojiao Ji, Jian Shen, Xinyi Lu

**Affiliations:** ^1^State Key Laboratory of Medicinal Chemical Biology, Nankai University, Tianjin 300371, China; ^2^College of Pharmacy, Nankai University, Tianjin 300350, China

## Abstract

Most of the human genome can be transcribed into RNAs, but only a minority of these regions produce protein-coding mRNAs whereas the remaining regions are transcribed into noncoding RNAs. Long noncoding RNAs (lncRNAs) were known for their influential regulatory roles in multiple biological processes such as imprinting, dosage compensation, transcriptional regulation, and splicing. The physiological functions of protein-coding genes have been extensively characterized through genome editing in pluripotent stem cells (PSCs) in the past 30 years; however, the study of lncRNAs with genome editing technologies only came into attentions in recent years. Here, we summarize recent advancements in dissecting the roles of lncRNAs with genome editing technologies in PSCs and highlight potential genome editing tools useful for examining the functions of lncRNAs in PSCs.

## 1. Introduction: Discovery of lncRNAs Expressed in Pluripotent Stem Cells

Pluripotent stem cells (PSCs) can unlimitedly self-renew and differentiate into specialized cell types of all three germ layers. Therefore, they have been used as an important *in vitro* model system for studying early development and generating an *in vivo* genome-edited animal model to analyze the physiological functions of a gene. Furthermore, they were used as a cell source for regenerative medicine to treat macular degeneration recently [1]. The two most frequently used types of PSCs are embryonic stem cells (ESCs), which are derived from inner cell mass of blastocyst, and induced pluripotent stem cells (iPSCs), which are established from somatic cells through reprogramming.

Long noncoding RNAs (lncRNAs) are >200 bp long RNA transcripts that lack coding capacity. Most of lncRNAs have evolved rapidly during evolution while a minority of lncRNAs are conserved through species [2]. The rapid evolution of lncRNAs could be partially explained by the presence of transposable elements in lncRNAs, since TEs are major contributors of lncRNA origination and diversification [3]. In the past two decades, expression arrays were first applied to identify novel lncRNAs [4–6]. Later, ENCODE Project Consortium and FANTOM Consortium used a high-throughput sequencing (HTS) method to identify novel transcripts from a genome [7, 8]. Currently, there are ~87,774 lncRNAs discovered in mouse cells and ~96,308 lncRNAs in human cells according to the NONCODE database [9]. Databases of lncRNAs have been established to catalogue novel lncRNAs and their functions (Table 1) [10, 11]. Given the importance of PSCs, further cataloguing of lncRNAs is essential for us to understand the complex regulatory network in PSCs. With the advancement of HTS technologies, ChIP-seq experiments revealed histone modifications as markers of gene transcription units. H3K4me3 was found to be a marker of promoter whereas H3K36me3 marked the gene body [12–14]. Therefore, H3K4me3 in combination with H3K36me3 could define the location of a transcribed gene [15]. With the application of histone marks to recognize discrete transcriptional units between protein-coding genes, the first genome-wide discovery of long intergenic noncoding RNAs (lincRNAs) was carried out in four cell types including embryonic stem cells (ESCs) [15]. The same study found that core pluripotency regulators Oct4, Sox2, and Nanog have driven the expression of lincRNAs, which in turn regulated the proliferation of ESCs [15]. These findings suggest lincRNAs as critical regulators in ESCs. Most of the discovered lncRNAs are functional [16]. They could participate in the regulation of multiple cellular processes such as transcription, RNA splicing, and translation regulation (Table 2) [17–19]. However, their exact roles in PSCs were mostly uncharacterized. Genome editing methods have been used extensively in probing the functions of protein-coding genes. It is a golden standard to demonstrate the role of a gene. However, the genome editing based on homologous recombination is inefficient, especially in human PSCs [20–23]. The introduction of molecular scissors, like the zinc finger nuclease (ZFN), transcription activator-like effector nuclease (TALEN), and clustered regularly interspaced short palindromic repeat (CRISPR/Cas9) systems, into this field allowed highly efficient genome editing in PSCs and other cell types [24–28]. These recent advances of genome editing technologies permit their more efficient and extensive usage in the analysis of lncRNA functions in PSCs. In this work, we review previous research into classic lncRNAs with genome editing and summarize the recent achievements in studying lncRNAs through novel genome editing technologies in PSCs.

## 2. Knockout lncRNAs in PSCs

The general strategy to study lncRNAs in PSCs is RNA interference (RNAi) mediated by short hairpin RNA (shRNA) or small interference RNA (siRNA). However, RNAi is unsuitable for a loss-of-function study of many lncRNAs. For instance, lncRNAs whose molecular functions are independent of the transcript, that is, the function of these lncRNAs, are from the transcription itself and not from the product of transcription. In addition, some lncRNAs, such as *MALAT1*, are highly abundant in the nucleus, so RNAi is inefficient in the depletion of these transcripts [62]. To study the physiological relevance of lncRNAs, their knockouts in ESCs or embryos are required to produce homozygous knockout animals. Therefore, it is critical to use genome editing for the loss-of-function study of lncRNAs in these cases to achieve the cleanest depletion of their expression.

### 2.1. Potential Challenges in the Generation of lncRNA Knockouts

**Figure 1:**
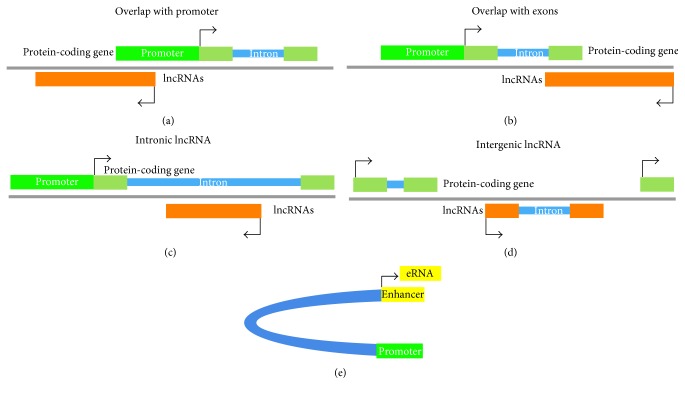
Location of lncRNAs on a human or mouse genome. (a) The lncRNA gene overlaps with the promoter of the protein-coding gene. (b) The lncRNA gene overlaps with exons of the protein-coding gene. (c) The lncRNA gene overlaps with the intron of the protein-coding gene. (d) The lncRNA gene is located between protein-coding genes. (e) lncRNAs, such as enhancer RNA, overlap with the enhancer region.

Molecular scissors have been used to create point mutations in the critical domains of protein-coding genes and in turn induce an early termination of translation to knock out a gene. Different from protein-coding genes, the functional domains of transcripts are still unclear for most lncRNAs; therefore, it is impossible to study lncRNAs by loss-of-function point mutagenesis. Thus, to knock out a lncRNA, complete or partial deletion of the lncRNA gene is required. To avoid indirect influences from lncRNA knockout, we need to manipulate lncRNA genomic loci without affecting the genomic features of other genes. However, under some circumstances, this is difficult to achieve. For lncRNAs that are located at the promoter region of other genes or overlap with exons of protein-coding genes (Figures 1(a) and 1(b)), partial deletion of them by genome editing should only be applied under the circumstances that the expression of other genes remains unaffected. For lncRNAs within the intron of the protein-coding gene, the deletion of lncRNA genes without disturbing the splicing of the intron region is required (Figure 1(c)). The lncRNAs at the intergenic region (Figure 1(d)), which are distant from other genes, could be easily removed by genome editing technology in a similar manner with protein-coding genes. However, intergenic lncRNA loci which overlap with enhancers (Figure 1(e)), such as enhancer RNAs, are also difficult to study with genome editing, because the deletion of these loci may interfere with the functions of enhancers and affect the expression of distant genes [63]. Therefore, in this section, we will discuss the knockouts of intergenic lncRNAs that do not overlap with enhancers.

### 2.2. Deletion of the lncRNA Gene

**Figure 2:**
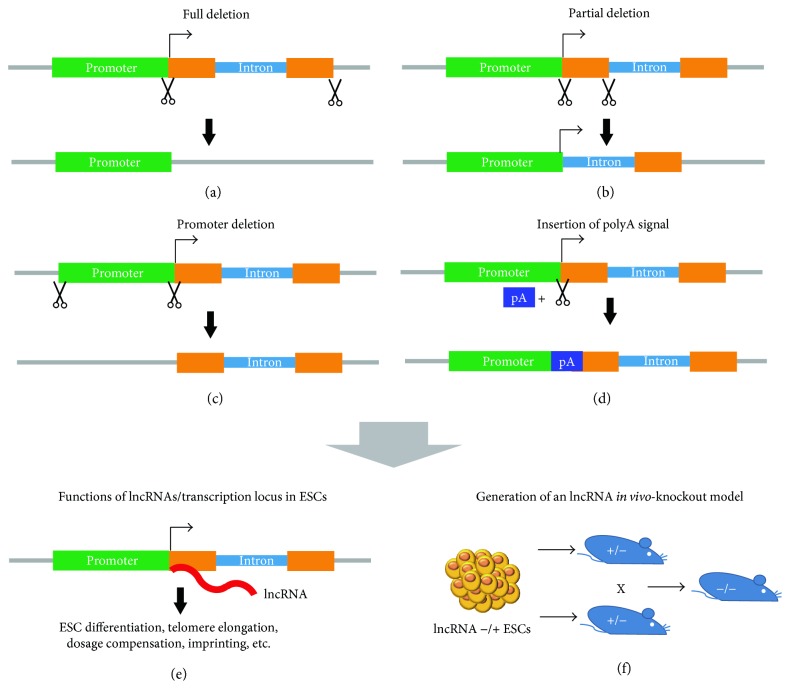
Application of the lncRNA knockout in PSCs. The lncRNA gene knockout can be done through (a) knocking out the whole lncRNA gene from the genome, (b) knocking out part of the lncRNA gene, (c) knocking out the lncRNA promoter region, and (d) inserting poly(A) signal (pA) after the transcription start site. (e) Application of genome editing to study lncRNA functions in ESCs. (f) Application of genome editing in ESC to generate lncRNA-knockout mice.

Whole-gene ablation of lncRNAs is a classic way to learn their functions (Figure 2(a)). Initial work on lncRNA knockouts was done in mouse ESCs but not in human ESCs, because of its early establishment and ease to be manipulated by homologous recombination [64]. ESCs have been used as a model to study imprinting, which occurs during ESC differentiation [65]. One of the first lncRNAs that have been identified and knocked out in mouse ESCs is the lncRNA *H19*. The homologous recombination-mediated deletion of maternal *H19* and its flanking sequences resulted in the expression activation of the imprinting gene *Igf2*, whereas the deletion of the paternal copy of *H19* has no impact on *Igf2* expression [66], suggesting *H19* as a lncRNA regulating maternal *Igf2* imprinting. The lncRNA *Terc*, a 397 bp RNA component of the telomerase complex, is another primary example of lncRNA knockout [67–69]. Ablation of the *Terc* lncRNA gene resulted in telomere shortening, which subsequently affected chromosome stability and proliferation of mouse ESCs [70, 71]. To improve the gene targeting efficiency, molecular scissors were introduced to engineer lncRNAs in PSCs. ZFNs were used in combination with homologous recombination to delete the highly expressed lncRNA *MALAT1* in mouse ESCs. From *MALAT1*-knockout ESCs, homozygous *MALAT1*-deleted mice have been generated [62]. TALEN was also used to knock out lncRNAs in zebrafish [72]. With the emergence of the CRISPR/gRNA system, the efficiency of genome editing is higher than before. It was demonstrated that double gRNAs could be applied to efficiently knock out lncRNAs in human cell lines [73]. Using the CRISRP/gRNA system, a full-length lncRNA (*HPAT5*) was successfully knocked out in human ESCs for the first time [59]. Recently, it is possible to delete the whole *H19* transcription unit and imprinting control region (ICR) by the CRISPR/Cas9 system in ESCs [74, 75]. This successfully restored *Igf2* expression and faithfully improved the efficiency to generate viable mice from androgenetic zygote and haploid ESCs [74, 75]. These studies demonstrate the complete deletion of lncRNAs through genome editing as an efficient way to discover lncRNA function in ESCs and during differentiation.

Certain lncRNAs are extremely long, so it is difficult to delete a full-length lncRNA gene. In these cases, partial deletion of the lncRNA gene through homologous recombination can be applied for the loss-of-function study (Figure 2(b)). The lncRNA *Xist*, discovered in the last century, was knocked out in this strategy. *Xist*, an ~18 kb lncRNA located on the X chromosome, functions as a central regulator of gene dosage compensation during ESC differentiation [76, 77]. Homologous recombination strategy was employed to delete part (~7 kb) of *Xist* in mouse ESCs [78], but the knockout efficiency is extremely low. More than 2500 clones were screened to identify a single homozygous knockout clone. Through this approach, *Xist* was found to be required for complete X chromosome inactivation during ESC differentiation [76, 77]. Heterogeneous knockout of *Xist* revealed a critical role of *Xist* for female embryo development [79]. Partial deletion of lncRNAs could also facilitate us to determine the function of RNA domains in lncRNAs. Through homologous recombination-mediated knockout in ESCs, an 890 bp region of the imprinting-related lncRNA *KCNQ1OT1* was found to be essential for *KCNQ1OT1* to recruit Dnmt1 protein to paternal differentially methylated regions [80, 81]. With the appearance of genome editing technology, megabase-scale genetic deletions with ZFN [82], TALEN [83, 84], or CRISPR [85] are achievable. The power of CRISPR technology in generating knockout of lncRNAs was demonstrated by the deletion of the lncRNA *Rian* in ESCs. A pair of sgRNAs in combination with CRISPR could delete 23 kb of the 57 kb *Rian* through zygote injection [86]. In addition, the knockout efficiency reached 33% if multiple sgRNAs were used [86]. This technology advancement may facilitate us to delete full-length extremely large lncRNAs in PSCs.

With the discovery of more lncRNAs and further understanding of lncRNAs' functions, the homologous recombination-based knockout of lncRNAs has been more extensively applied to delete lncRNAs in PSCs. In one of the studies, 18 lncRNA genes were knocked out in mouse ESCs to produce lncRNA-knockout mice [87]. The replacement of the lncRNA locus with the *lacZ* reporter allowed the visualization of the temporal and spatial expression pattern of these lncRNAs in animal models. Another similar study created knockout ESC lines for 20 lncRNAs through gene targeting and used these ESCs to create knockout mice for studying the broad roles of lncRNAs in mice [88]. These lncRNA-knockout mice constitute valuable complements to the resource for studying the physiological roles of lncRNAs.

### 2.3. Knocking In Polyadenylation Signal

Another strategy to prevent lncRNA transcript production is the knockin of polyadenylation (polyA) signal at the transcription start sites (TSS) (Figure 2(d)). Biallelic insertion of one copy or multiple copies of polyA signal at the beginning of lncRNA gene TSS will cause early termination of transcription and the subsequent failure of lncRNA production [89]. However, for lncRNAs with alternative promoters and transcription TSS, this strategy may not be applicable. This strategy was employed in ESCs to characterize the functions of the lncRNA *Fendrr* in embryo development [90, 91]. PolyA insertion-mediated *Fendrr* knockout led to malfunctioned heart and embryonic death by E13.75 in mice, while overexpression of *Fendrr* through BAC rescued the phenotype. Moreover, this method prevents lncRNA production without disturbing the transcription activity itself. Hence, this approach allows the distinguishment of the function of the lncRNA itself from that of its genomic transcription activity. This is exemplified by the case of the imprinting lncRNA *Airn*. The *Airn* gene overlaps with *Igf2r* promoter regions and is transcribed in the opposite direction of *Igr2r* [92]. The *Airn* gene spans more than 100 kb of the mouse genome and encodes multiple spliced isoforms [92]. Expression of *Airn* on paternal allele represses the paternal expression of *Igf2r*, *Slc22a3*, and *Slc22a2* during ESC differentiation [92, 93]. Surprisingly, truncation of *Airn* by insertion of polyA signals after TSS did not affect *Igf2r* gene expression [94]. In addition, the overlapped transcription at the *Airn* promoter region is sufficient to repress *Igf2r* after ESC differentiation [94]. Furthermore, the *Airn* gene is very large (>100 kb) and may be difficult to knock out. This study also demonstrates that polyA signal could be a more efficient way to prevent expression of macro lncRNA genes than whole-gene deletion.

### 2.4. Deletion of the lncRNA Promoter

Promoters of lncRNAs are critical to drive their expression. For intergenic lncRNAs, another strategy to disrupt their expression relied on the removal of the lncRNA promoter by genome editing (Figure 2(c)). With two gRNAs expressed simultaneously, the promoter of lncRNAs could be efficiently deleted to achieve silencing of lncRNA expression [95]. One example is from the classic lncRNA *H19*, whose knockout allows the derivation of bimaternal mice [96, 97]. Similar to *H19* knockout, deletion of DMRs of lncRNAs *H19* and *Gtl2* in haploid ESCs by CRISPR represses *H19* and *Gtl2* expression and allows the generation of semicloned mice from haploid ESCs [16]. These suggest the deletion of the lncRNA promoter region as an efficient approach to silence lncRNA expression in PSCs.

### 2.5. Combination of Different lncRNA Knockout Methods

The above examples show that a single approach is insufficient to identify the all possible functions of lncRNAs. Multiple genome editing strategies need to be taken in order to discover the functions of lncRNAs and their transcription locus in PSCs. This is well demonstrated in the study of *Haunt* lncRNAs in ESCs. Yin et al. found that siRNA mediated *Haunt* lncRNA depletion and deleting a small fraction of the *Haunt* gene caused a consistent further increment in *HOXA* expression upon retinoic acid- (RA-) induced ESC differentiation [63]. However, large deletions of the *Haunt* gene ranging from 7.3 kb to 58 kb caused an opposite effect. Disruption of *Haunt* expression by CRISPR/Cas9-mediated knockout of 2.3 kb of the *Haunt* promoter region or insertion of 4 × polyA signal after transcription start sites also leads to enhanced activation of *HoxA* cluster genes after ESC differentiation. These observations demonstrate distinct roles of lncRNAs and their corresponding genomic locus in regulating RA-induced *HOXA* expression. Moreover, this study also implicates that the role of lncRNAs should be examined in different aspects and multiple approaches to reveal the functions of the lncRNA gene locus and its transcripts in PSCs (Figures 2(e) and 2(f)).

## 3. lncRNA Reporter Gene in PSCs

**Figure 3:**
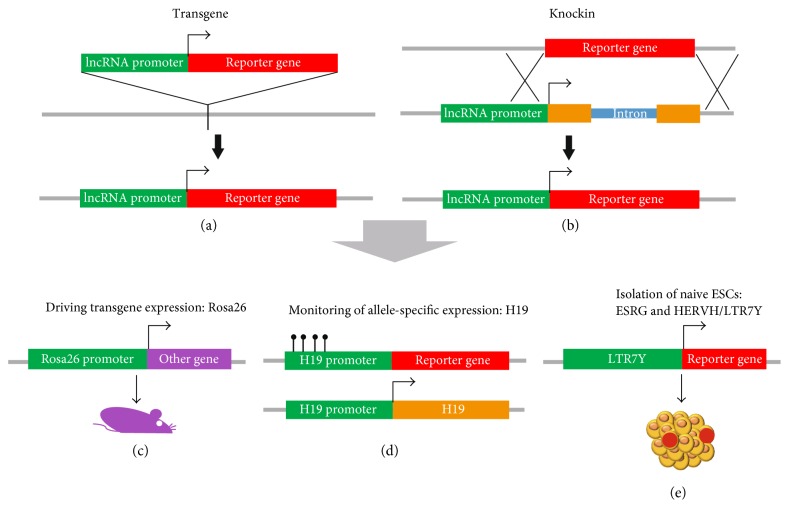
Application of the lncRNA reporter gene in PSCs. Generation of lncRNA reporter genes can be achieved by (a) knocking in the reporter gene to the lncRNA locus and (b) introducing the lncRNA promoter-driven reporter gene. Application of the lncRNA reporter gene in (c) driving the expression of the transgene (such as the *Rosa26* locus). (d) Monitoring the allele-specific gene expression. (e) Isolation of naive ESCs (red dot) (such as the *ESRG* promoter- or LTR7Y-driven transgene).

Creation of the lncRNA reporter gene in PSCs allows us to track lncRNA expression *in vivo* and study the regulation of lncRNAs. In order to create reporter genes of lncRNAs, the differences between lncRNAs and protein-coding genes have to be considered. Unlike protein-coding genes, lncRNAs do not encode proteins. For this reason, it is impossible to make fluorescent fusion proteins through addition of self-cleaving 2A peptide or introduction of internal ribosome entry sites (IRES) to create the lncRNA reporter gene. The addition of protein-coding gene sequences to lncRNAs may interfere the localization and function of lncRNAs, whereas the inclusion of IRES sequence may lead to the recruitment of ribosomes to lncRNAs and conversion of lncRNAs to mRNAs. Therefore, the creation of lncRNA promoter-driven reporters requires genome editing of lncRNAs by knocking in the lncRNA locus or introducing an independently expressed transgene to the genome (Figures 3(a) and 3(b)). However, knocking in the reporter gene to the lncRNA locus will destroy one copy of the lncRNA gene and affect the expression of neighboring genes if the lncRNA acts in cis. Introduction of the lncRNA promoter-driven transgenic reporter may not reflect the true expression pattern of lncRNAs because the usage of enhancers and silencers is different at distinct genomic loci. All these situations need to be considered prior to the establishment of the lncRNA reporter PSC cell line.

The earliest application of the lncRNA reporter in PSCs is to express foreign genes at the lncRNA transcription locus. A primary instance is the *Rosa26* locus, which is used to constitutively overexpress genes for ~20 years. The *Rosa26* locus was first discovered in 1991 during gene trapping in ESCs [98]. Later, it was found to encode two nuclear transcripts with no significant open reading frames (ORFs), suggesting them as lncRNAs [99]. Interrupting this locus with the proviral beta geo reporter gene led to ubiquitous expression of beta-galactosidase, suggesting that the *Rosa26* locus encodes universally expressed lncRNAs in mice [99]. Since then, the *Rosa26* locus has been used as a genetic safe harbor for gene knockin to achieve ubiquitous transgene expression [100, 101] (Figure 3(c)). Nowadays, the human *Rosa26* locus was also discovered, and numerous genes have been knocked in the *Rosa26* locus in ESCs to generate knockin mice and study the function of these genes [101, 102].

lncRNAs are involved in the essential gene regulatory processes during development. The reporter system of lncRNAs is also used to monitor the regulatory status of important biological processes in ESCs and during differentiation. One instance is using the paternal *H19* reporter gene to monitor imprinting status during ESC differentiation. Since *H19* expression is essential to maternal imprinting, to avoid the interruption of the *H19* gene, the transgene carrying *H19* promoter-driven *lacZ* and *PLAP* was used as the reporter to reflect the change of imprinting status [103] (Figure 3(d)). Using this reporter system, 1.1 kb control element was discovered to regulate maternal *H19* imprinting [103]. A recent example of the application of the lncRNA reporter is to isolate naive human ESCs. Endogenous retrovirus HERVH was discovered as ESC-specific lncRNAs that regulate pluripotency [17]. The HERVH promoter (LTR7Y) is active only in cells from inner cell mass of blastocyst. Thus, the transgenic LTR7Y-driven GFP reporter can be used for the isolation of naive-state human ESCs [104] (Figure 3(e)). It was also found that the reporter gene driven by the promoter of the HERVH-derived lncRNA *ESRG* marked naive human ESCs [105] (Figure 3(e)). All preceding examples demonstrate the power of lncRNA reporter genes in studying the regulation of lncRNAs and tracking their expression.

## 4. Activation and Repression of lncRNAs with Genome Editing Technologies

**Figure 4:**
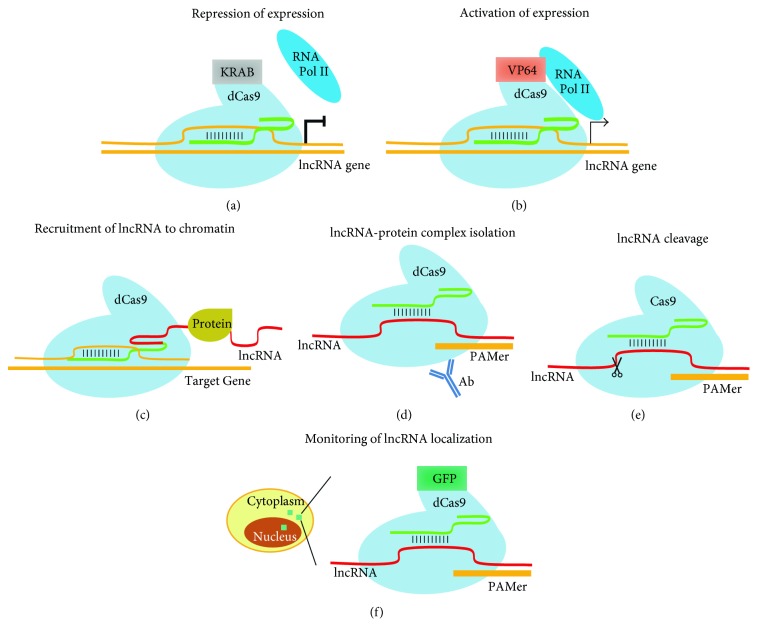
Potential applications of the CRISPR/Cas9 system to study lncRNAs in PSCs. Application of CRISPR/Cas9 technology in the (a) repression of lncRNA transcription, (b) activation of lncRNA expression, (c) recruitment of the lncRNA to chromatin, and (d) isolation of the lncRNA-protein complex. (e) Degradation of the lncRNA. (f) Monitoring lncRNA localization. Green strand: gRNA.

Since CRISPR/Cas9-based genome editing technology could efficiently delete large fragments of the genome [86], it has been utilized to perform genome-wide screening of lncRNA functions [106]. Multiple gRNAs were used against one single lncRNA to accomplish efficient ablation of the lncRNA gene expression; therefore, the paired gRNA library could only target a few hundreds of lncRNAs [106]. Using this approach, lncRNAs critical to cancer cell survival have been identified. An alternative approach to modulate lncRNAs with CRISPR-Cas9 is through CRISPR interference (CRISPRi) [107], which is constituted of deactivated Cas9 (dCas9) fused with transcription repressors, such as KRAB. Through the recruitment of dCas9-KRAB to the promoter regions of lncRNAs with multiple gRNAs, the expression of lncRNAs is hindered by the transcription repressors recruited by KRAB protein (Figure 4(a)). CRISPRi was applied to manipulate lncRNA (*GAS5*, *H19*, *MALAT1*, *NEAT1*, *TERC*, and *XIST*) expression in K562 cells [108]. In addition to these lncRNAs, CRISPRi was applied to probe the function of the cheRNA *HIDALGO* in K562 and H1 human ESCs [109]. This was adapted at a genome-wide scale to perform lncRNA repression screen in various cell types including human induced pluripotent stem cells (iPSCs) [110]. In this way, a number of lncRNAs were discovered as self-renewal regulators of human iPSCs [110]. The efficiency of genome editing is regulated by the epigenetic status of chromatin, such as chromatin conformation. The genome editing efficiency with TALEN and CRISPR is higher for genes at euchromatin than at heterochromatin [111]. This may introduce bias to genome-scale CRISPR-mediated screening of gene expression regulators.

Modified CRISPR is applicable not only for depleting gene expression but also for activating gene expression. CRISPR activation (CRISPRa), which utilizes dCas9 fused with multiple copies of strong viral transcription activators such as VP16, activates gene expression by bringing RNA polymerase II to TSS [107, 112]. This method can be adopted to activate lncRNA expression in ESCs (Figure 4(b)). Traditionally, in terms of the gain-of-function study of lncRNAs, plasmid- or transgene-based overexpression of lncRNAs was used. However, different from protein-coding genes, some lncRNAs function during transcription, that is, the production of transcript [63, 94]. Therefore, exogenous expression of lncRNAs may not reflect the genuine function of lncRNAs. In addition, multiple isoforms are present for some lncRNAs [94]. It is difficult to overexpress all isoforms at the same time. What is more, for some lncRNAs that lack polyA tail or are derived from introns [113], it is important to clone the fraction longer than its expression region. Moreover, for the lncRNA acting in cis, introduction of the lncRNA transgene to other genomic locations for overexpression cannot reflect the true function of lncRNAs. Above difficulties in lncRNA overexpression could be conquered by the usage of CRISPRa to activate the lncRNA. Recently, Joung's group applied CRISPRa in a genome-wide scale to identify lncRNAs that render melanoma cells' drug resistance to vemurafenib [114]. CRISPRa directly activates the expression of endogenous genes from their genomic locus [107], and therefore, it keeps the function of the lncRNA transcriptional region and makes the simultaneous activation of multiple lncRNA isoforms possible. These methods could be applied to PSCs for the gain-of-function study of the lncRNA regulators of pluripotency maintenance.

## 5. Other Potential Applications of Genome Editing Tools in Studying lncRNAs in PSCs

CRISPR/Cas9 is a versatile tool for genome editing and expression regulation. Besides its applications in editing the genetic locus in ESCs, CRISPR/Cas9 can be adopted to investigate other aspects of lncRNA biology in ESCs. A number of nuclear lncRNAs may act by interacting with chromosomes to regulate gene expression [115, 116]. The inhibition of expression of these lncRNAs by CRISPR/Cas9-mediated truncation of their promoters will cause downregulation of neighboring genes' expression [109, 117]. To study the role of these lncRNAs, a valuable tool, named CRISPR-Display, was developed to deliver an lncRNA-protein complex to DNA loci [118]. Functional RNA domains can be inserted into gRNAs, allowing the identification of the direct effect of ectopically targeting lncRNAs on chromatin (Figure 4(c)). In addition, this system can be multiplexed to investigate the influences of recruitment of lncRNAs on several genomic loci simultaneously. Moreover, it was recently discovered that the CRISPR system could be edited to interact with cellular RNAs. Cas9 directly binds or cuts RNAs in the assistance of DNA PAMmers [119]. This enables the cleavage of RNA or pulldown of mRNA through RNA-RNA hybridization by Cas9-gRNA [119]. It can be used to cleave lncRNAs with Cas9 or pull down lncRNAs with dCas9 in ESCs to analyze the potential interacting proteins of lncRNAs (Figures 4(d) and 4(e)). With this system, dCas9 fused with GFP is targeted to mRNAs to track their localization in live cells with the guidance of sgRNA [120]. This system can also be adopted to study lncRNA position in PSCs and track lncRNA localization in live PSCs (Figure 4(f)).

## 6. Conclusions and Perspectives

In the recent years, thousands of lncRNAs have been identified. Several of them were shown to play important roles in PSCs [116]. However, the advancements in genome editing technologies are just starting to be widely applied in PSCs to study the functions of lncRNAs. Considering the diverse functions of the lncRNA genomic locus and its transcript(s), multiple genome editing approaches should be applied to distinguish the functions of the lncRNA transcript and its gene locus in PSCs. lncRNAs are important biomarkers in embryo development and disease progression. The establishment of the lncRNA reporter *in vivo* will enable the monitoring of these processes. The development of emerging CRISPR genome editing technologies opens new gates to lncRNA biology in PSCs. Future studies should adopt these novel strategies to probe the functions of lncRNAs in PSCs. These genome editing tools should also be exploited to explore physiological functions of lncRNAs in a systematic scope.

## Figures and Tables

**Table 1:** Introduction of different lncRNA databases.

Name	Date	Species	Function	Website	Ref.
Comprehensive annotations of lncRNAs					
NONCODE	2005	17 species	Gene function annotation	http://www.noncode.org/	[9]
lncRNAdb	2011	68 species	Comprehensive annotations of functional lncRNAs	http://www.lncrnadb.org/	[29]
lncRNome	2013	Human	Integrating annotations on a wide variety of biologically significant information	http://genome.igib.res.in/lncRNome/	[30]
lncRNAtor	2014	6 species	Functional investigation of lncRNAs	http://lncrnator.ewha.ac.kr/	[31]
LncRNAWiki	2015	Human	Comprehensive integration of information on human lncRNAs	http://lncrna.big.ac.cn	[32]
Annotation of lncRNA interactions					
LNCipedia	2013	Human	Annotation of lncRNA transcript sequences and structures	https://www.lncipedia.org	[33]
Linc2GO	2013	Human	lincRNA function annotation based on ceRNA hypothesis	–	[34]
Starbase	2014	Human, mouse, and *C. elegans*	Annotation of miRNA-lncRNA/mRNA interactions	–	[35]
NPInter	2014	18 species	Interactions between ncRNAs and biomolecules	http://www.bioinfo.org/NPInter	[36]
lncACTdb	2015	Human	lncRNA-miRNA-gene interactions	–	[37]
Transcriptional regulatory networks of lncRNAs					
ChIPBase	2013	6 species	Transcriptional regulatory networks of ncRNAs and PCGs	–	[38]
SNP@lincTFBS	2014	Human	Annotation of SNPs in potential TFBSs of lincRNAs	http://bioinfo.hrbmu.edu.cn/SNP_lincTFBS	[39]
TF2LncRNA	2014	Human	Identifying common transcription factors of lncRNAs	–	[40]
lncRNA-associated pathways					
LncReg	2015	Human and mouse	lncRNA-associated regulatory networks	http://bioinformatics.ustc.edu.cn/lncreg/	[41]
Co-LncRNA	2015	Human	Investigating the lncRNA combinatorial effects in GO annotations and KEGG pathways	http://www.bio-bigdata.com/Co-LncRNA//lncreg/	[42]
lncRNA-disease associations					
C-It-Loci	2015	Human, mouse, and zebrafish	Tissue-specific lncRNAs	http://c-it-loci.uni-frankfurt.de	[43]
LncRNADisease	2013	Human	lncRNA-disease associations	–	[44]
lnCaNet	2016	Human	lncRNA-cancer gene coexpression network	http://lncanet.bioinfo-minzhao.org/	[45]
Lnc2Cancer	2016	Human	Exploring lncRNA deregulation in various cancers	http://www.bio-bigdata.net/lnc2cancer	[46]

**Table 2:** Classification of lncRNA functional mechanisms and the location of lncRNAs.

Mechanism of function	Examples	Ref.
Nucleus		
Regulating chromatin-modifying complexes	*ANRIL*	[47]
	*HOTAIR*	[48]
Recruiting transcription factors	*HERVH*	[17]
	*PWR1*	[49]
	*TUNA*	[50]
Chromatin remodeling	*HOTTIP*	[51]
	*SRG1*	[52]
	*fbp1 ncRNA*	[53]
Influencing pre-mRNA splicing	*MALAT1*	[18]
	*EGFR-AS1*	[54]
Cytoplasm		
Regulating mRNA stability	*Linc-RoR*	[55]
	*Gadd7*	[56]
	*MEG3*	[57]
Regulating mRNA translation	*lincRNA-p21*	[19]
	*Antisense Uchl1*	[58]
Competing for microRNA binding	*HPAT5*	[59]
	*HULC*	[60]
Translated in biologically active small peptides	*LINC00961*	[61]
